# 

**Published:** 1895-08-31

**Authors:** 


					ADoui^u i c.lh?r u kj;., Liiereiore alibi. ^uuust am", 159a,
OADBURY'S ww CADBURY'S
f i|
?* or 1 "
a S0'"te purity and freedom from alkali." " The Typical Cocoa of English Manufacture
?Braithwaite. ?Absolutely Pure."? The Analyst.
[?ASConr Wes TERN I UFIRMARt^"^"~ ^?~= -? 'fidftf.OLjiA.ua NORWICH HQSflJAi.
C0; 466^ ^oL xvm- WITH EXTRfl NURSING supplement. *g?2L.'EZ?*??S*'
wDAv August 31st 189*1 [[Registered at the General Post Office as a Newspaper for Monthly Parts, 6d.; post free, 8d.
^***^ ? ' transmission in the United Kingdom and Abroad.] Ditto per Annnm,8s.6d.,poBt free.

				

